# The Effects of Soil Depth on the Structure of Microbial Communities in Agricultural Soils in Iowa (United States)

**DOI:** 10.1128/AEM.02673-20

**Published:** 2021-01-29

**Authors:** Jingjie Hao, Yen Ning Chai, Lucas Dantas Lopes, Raziel A. Ordóñez, Emily E. Wright, Sotirios Archontoulis, Daniel P. Schachtman

**Affiliations:** a Department of Agronomy and Horticulture, University of Nebraska—Lincoln, Lincoln, Nebraska, USA; b Center for Plant Science Innovation, University of Nebraska—Lincoln, Lincoln, Nebraska, USA; c Department of Agronomy, Iowa State University, Ames, Iowa, USA; d Department of Plant Science and Industries Building, Pennsylvania State University, University Park, Pennsylvania, USA; University of Michigan—Ann Arbor

**Keywords:** Iowa, USA, agricultural soils, microbial communities, soil depth

## Abstract

This study investigated the differences in microbial community abundance, composition, and diversity throughout the depth profiles in soils collected from corn and soybean fields in Iowa (United States) using 16S rRNA amplicon sequencing. The results revealed decreased richness and diversity in microbial communities at increasing soil depth. Soil microbial community composition differed due to crop type only in the top 60 cm and due to location only in the top 90 cm. While the relative abundance of most phyla decreased in deep soils, the relative abundance of the phylum *Proteobacteria* increased and dominated agricultural soils below the depth of 90 cm. Although soil depth was the most important factor shaping microbial communities, edaphic factors, including soil organic matter, soil bulk density, and the length of time that deep soils were saturated with water, were all significant factors explaining the variation in soil microbial community composition. Soil organic matter showed the highest correlation with the exponential decrease in bacterial abundance with depth. A greater understanding of how soil depth influences the diversity and composition of soil microbial communities is vital for guiding sampling approaches in agricultural soils where plant roots extend beyond the upper soil profile. In the long term, a greater knowledge of the influence of depth on microbial communities should contribute to new strategies that enhance the sustainability of soil, which is a precious resource for food security.

**IMPORTANCE** Determining how microbial properties change across different soils and within the soil depth profile will be potentially beneficial to understanding the long-term processes that are involved in the health of agricultural ecosystems. Most literature on soil microbes has been restricted to the easily accessible surface soils. However, deep soils are important in soil formation, carbon sequestration, and providing nutrients and water for plants. In the most productive agricultural systems in the United States where soybean and corn are grown, crop plant roots extend into the deeper regions of soils (>100 cm), but little is known about the taxonomic diversity or the factors that shape deep-soil microbial communities. The findings reported here highlight the importance of soil depth in shaping microbial communities, provide new information about edaphic factors that influence the deep-soil communities, and reveal more detailed information on taxa that exist in deep agricultural soils.

## Abstract (precis)

Determining how microbial properties change across different soils and within the soil depth profile will be potentially beneficial to understanding the long-term processes that are involved in the health of agricultural ecosystems. Most literature on soil microbes has been restricted to the easily accessible surface soils.

## INTRODUCTION

Microbial communities play pivotal roles in the ecosystem, plant and animal health, food safety, and crop production (1–3). Soils are one of the most diverse microbial ecosystems on Earth, containing microscopic bacteria and fungi, microfauna (nematodes and protozoans), mesofauna, and macrofauna (4). Soil microbiomes are a foundational feature of the agricultural ecosystems and host various biogeochemical processes, such as nutrient cycling and decomposition of organic matter (5). A primary role of soils is to provide plant roots with the nutrients required for growth and productivity. Soil microbial communities coexist with roots in the plant-microbe-soil system (6), and the structure of microbial communities strongly influences critical processes required for plant growth, such as nitrogen cycling and organic matter decomposition (7–9).

Despite the extensive presence of soil microbes throughout the soil profile, our current understanding of the diversity and composition of soil microbial communities is mainly restricted to surface soils (0 to 25 cm), where there tend to be higher levels of soil nutrients and organic matter and a higher diversity of microorganisms than in the subsurface layers (10, 11). In agricultural production systems, plant roots grow below 25 cm and the deeper soils are important for crop yield, because topsoils may dry out quickly during summer months, limiting the ability of roots to absorb water and nutrients in the upper layers of the soil profile. Therefore, extending our knowledge about microbial community structure to deeper depths is critical.

The composition of soil microbial communities is influenced by habitat types and a variety of edaphic factors, such as soil pH, texture, moisture, mineral nutrient content, and organic matter (12–17). Previous studies have demonstrated remarkable changes in microbial community composition with soil depth across different environments (11, 18–21), such as consistent decreases in microbial abundance and diversity with deep soils (down to 2 m) (22). Comparisons of microbial community composition between surface soils and subsurface soils have revealed drastic differences in soil nutrients, extracellular enzyme activities, soil organic carbon (C), and microbial biomass (23, 24). However, a majority of studies focused on the microbial diversity between the surface and subsurface soils to a depth of 100 cm in nonagricultural soils or in very specialized environments. The deeper soil microbial communities are very important to characterize because they have greater impact on soil-forming processes than surface soils (25). In addition, deep soils comprise, on average, greater than 50% of the total soil organic carbon, and so microbial processes down deep (2 to 3 m) are important to understand because of their roles in carbon sequestration (26). Therefore, exploring the characteristics of subsurface soil microbial communities throughout the soil profile will eventually enable a better understanding of multiple soil processes, which play a role in contributing to the productivity of the agroecosystem (27).

In addition to soil depth, plant roots are another key factor that influence soil microbial activities in various ecosystems (28, 29). Since plant growth is dependent on edaphic factors, the plant-soil interactions at different soil depths play a role in the abundance and composition of soil microbial communities (30). Although most studies have focused on nutrient rich topsoil, the roots of agricultural crops can grow as deep as 200 cm (31). For example, the average maximum rooting depth of corn and soybean grown in the midwestern United States is 150 cm (32). Although soil depth shapes soil microbial community composition in arable soil (33), it is not known how roots shape communities in deep soils. Changes in key bacterial taxa that utilize plant-derived carbon in the rhizosphere of wheat at different depths in soil have been reported (34). Investigating the soil microbial community structure along the depth of crop rooting systems, especially in deeper soil profiles, will provide insights into distinct and potentially important processes involved in agricultural soil nutrient cycling and long-term carbon storage (35).

A detailed understanding of the soil microbial properties with respect to changes in soil depth will potentially contribute to the long-term health of agricultural soils or the diagnosis of unhealthy soils. This study was carried out with soils collected from corn and soybean fields in Iowa, which were located in one of the world’s most productive agricultural regions (36). A 16S rRNA amplicon data set of soil DNA from seven different depths was sequenced on an Illumina MiSeq platform. The objective was to investigate the effects of depth on the microbial community abundance, composition, and diversity throughout the soil profile in these agricultural fields. This study sought to answer the following questions. (i) Are soil microbiomes in agricultural fields strongly affected by soil depth? (ii) How do other soil properties besides depth influence soil microbial community composition? (iii) What are the changes in specific microbial taxa along soil depth profile?

## RESULTS

### Decreased richness and diversity in microbial community along soil depth gradient.

**FIG 1:**
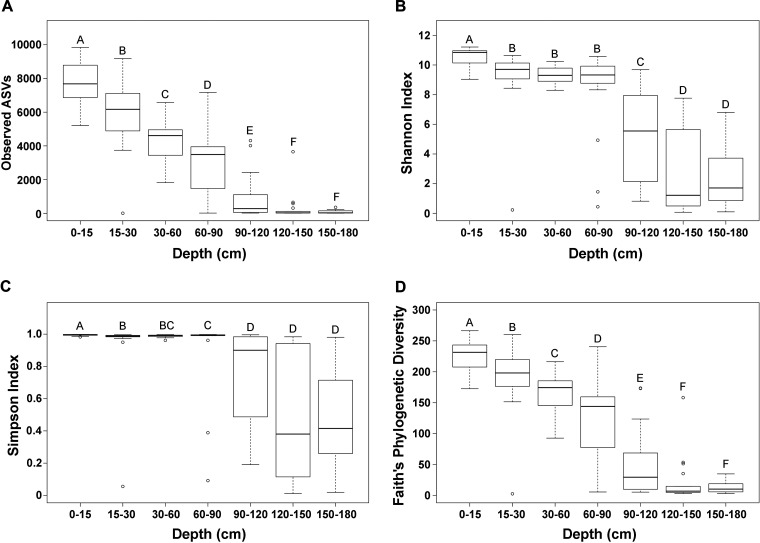
Changes in alpha diversity levels with soil depth. (A) Average number of observed ASVs at different soil depths. (B) Shannon index at different soil depths. (C) Simpson index at different soil depths. (D) Faith’s phylogenetic diversity index at different soil depths. Differences in alpha diversity were compared using Wilcoxon test adjusted for false-discovery rate. A *P *value of <0.05 was considered statistically significant. Different letters above the bars indicate significant differences between soil depths. Lines in boxes represent medians. The top and bottom of each box represent the first and the third quartiles, respectively. Whiskers indicate data ranges, with outliers shown as open circles.

Microbial species richness as determined by observed amplicon sequence variants (ASVs) was highest in the surface soil and significantly decreased as soil depth increased, but the richness of the microbial community was not significantly different between 120 and 150 cm and 150 to 180 cm (Fig. 1A). The diversity of the microbial community as determined by the Shannon index was significantly different between 0 and 15 cm and 15 to 30 cm, but not significantly different between 15 and 90 cm, and significantly decreased between 90 and 180 cm (Fig. 1B). The Simpson index of species diversity was significantly different between the upper soil layers (0 to 90 cm) and the deep soil layers (90 to 180 cm) (Fig. 1C). Furthermore, the microbial communities were significantly distinct along the soil depth profile as determined by the Faith’s phylogenetic diversity, with the exception of 120 to 150 cm and 150 to 180 cm (Fig. 1D). Alpha diversity indices at different sites and crops at each individual depth were also investigated, and no significant difference was detected between different sampling sites (see Fig. S1 in the supplemental material) or between different crop types (Fig. S2) along the soil profile.

### Soil depth shifts the microbial community composition and bacterial abundance.

**FIG 2:**
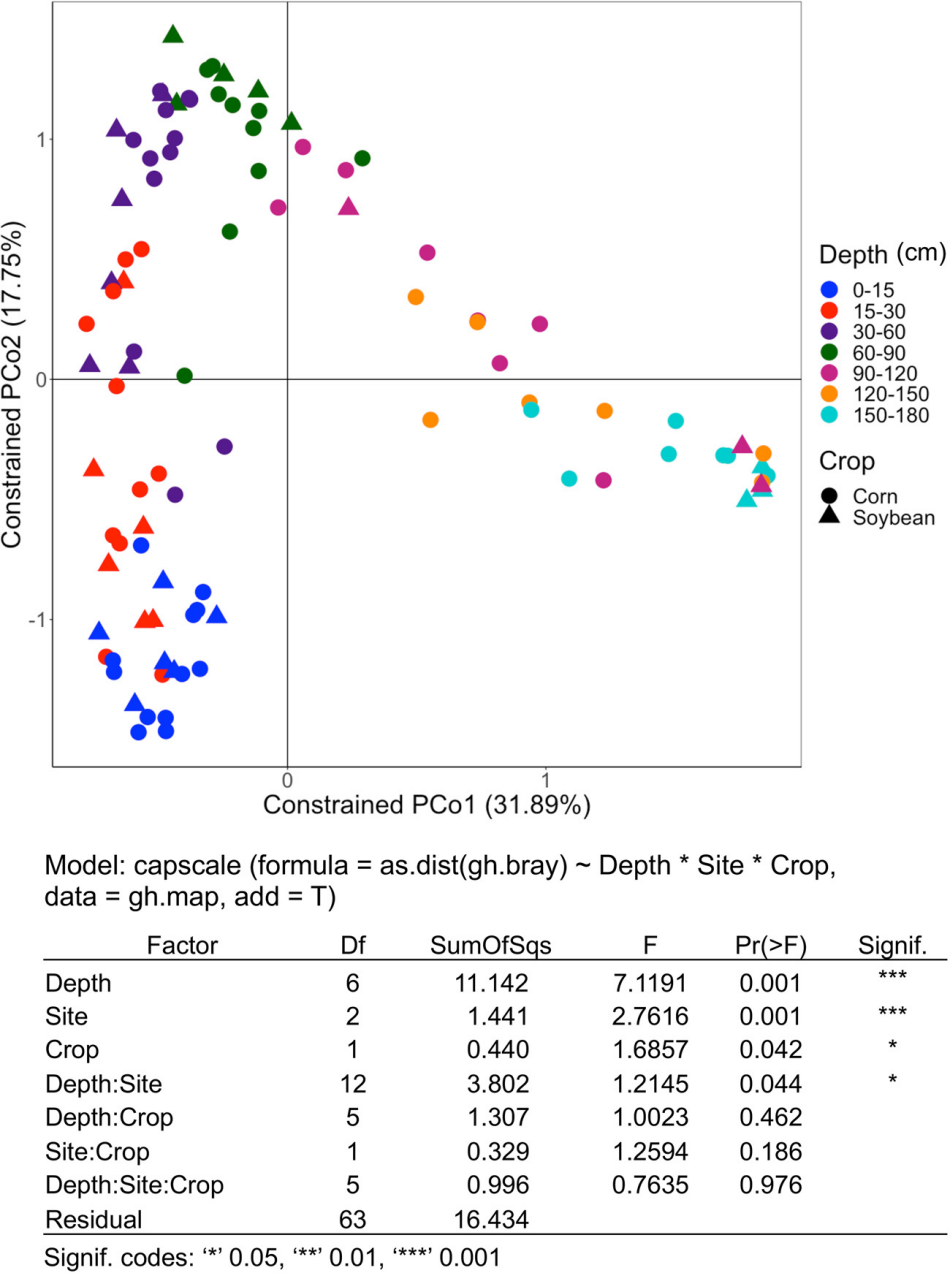
Beta diversity showing changes in microbial community composition with depth, site, and crop type. Canonical analysis of principal coordinates (CAP) using Bray-Curtis dissimilarity for all samples was conducted. The Bray-Curtis dissimilarity matrix was generated using QIIME. CAP was conducted by constraining soil depth, crop type, and sampling site using the ‘capscale’ function in the vegan R package. PERMANOVA was performed to determine whether the shifts in microbial community due to soil depth, crop type, and sampling site and their interactions were significant. Each color indicates different soil depth as shown in the key.

**FIG 3:**
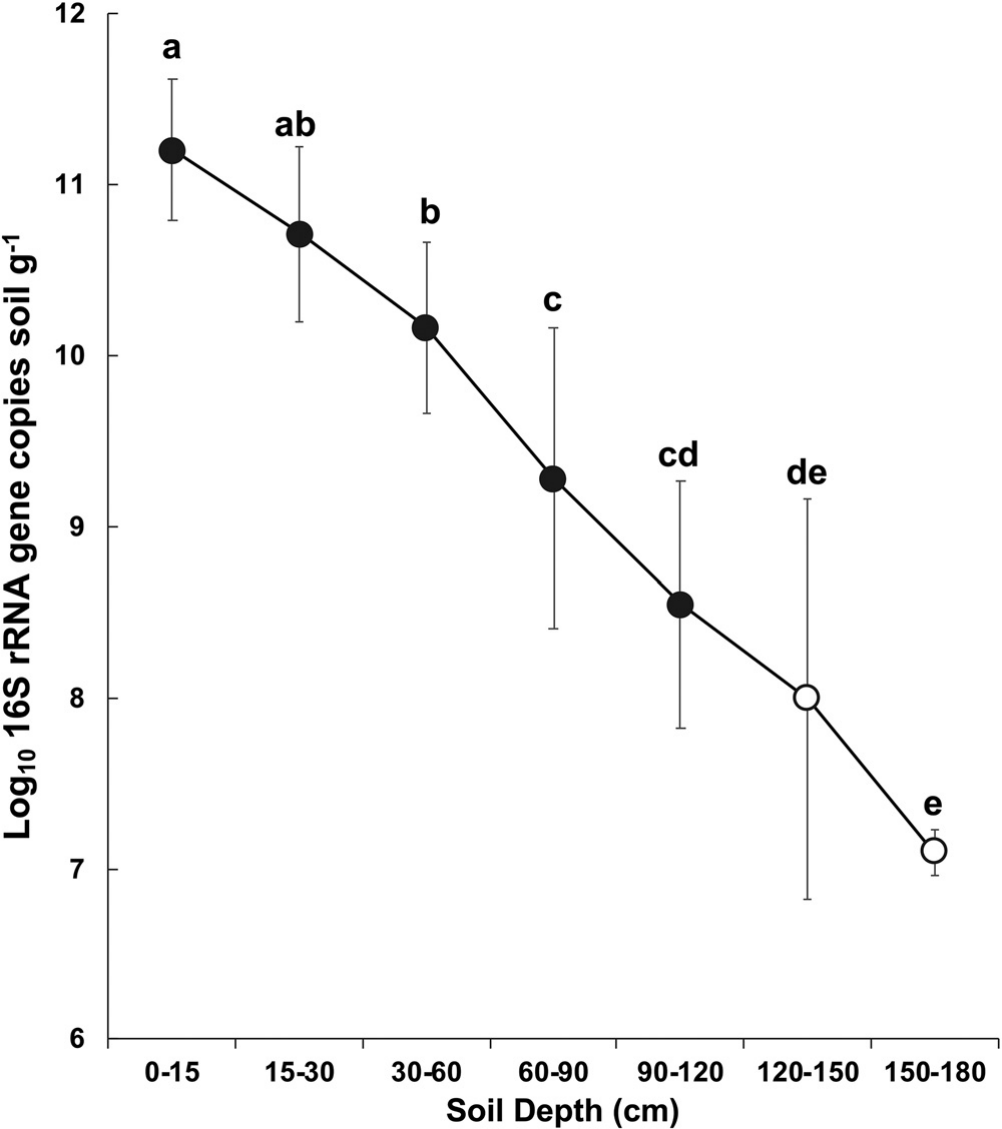
Bacterial abundance as determined by 16S rRNA gene copy number at different soil depths. Quantitative PCR results show the average 16S rRNA gene copies per gram of soil at each soil depth. The open symbols indicate that at specific depths many samples were below the detection level for the standard curve and were not included in the averages. Three and 4 out of 18 samples were used for calculating the average copy number at 120 to 150 cm and 150 to 180 cm, respectively.

Canonical analysis of principal coordinates (CAP) was performed to evaluate how each factor in the data set, including soil depth, sampling site, and crop type, contributed to the variation in microbial community composition. Soil microbial community composition shifted significantly with soil depth (*P < *0.001, 31.0% variation explained), sampling sites (*P < *0.001, 4.0% variation explained), and crop types (*P < *0.05, 1.2% variation explained) (Fig. 2). In addition, there was a significant interaction between depth and site (*P < *0.05, 10.6% variation explained) (Fig. 2). To assess the influence of soil depth alone on soil microbial community composition, CAP was performed based on a Bray-Curtis dissimilarity matrix, factoring out the effect of site and crop type. It revealed that the microbial community composition was significantly different among samples at different soil depths (*P < *0.001) (Fig. S3). The ordination showed some separation along the first axis of the microbial communities in the upper (0 to 90 cm) versus the deeper (90 to 180 cm) soils. The greater apparent separation of samples at the different depths along the second axis of the ordination in the top 0 to 90 cm of the soil profile suggests more heterogeneity in microbial community composition in the upper profile than in the deeper profile (90 to 180 cm) (Fig. S3). CAP was also conducted using both weighted UniFrac (WUF) and unweighted UniFrac (UUF) distance metrics, and soil depth also influenced the soil microbiome (Fig. S4). In addition to shaping the structure, soil depth also significantly affected the abundance of bacterial communities estimated by 16S rRNA gene quantification, which decreased exponentially with soil depth (Fig. 3). A total of 83 samples showed values higher than the lowest concentration of the standard curves. Samples ranged from a minimum average value of 1.25 × 10^7^ (150 to 180 cm) to a maximum average value of 1.59 × 10^11^ (0 to 15 cm) 16S rRNA gene copies g^−1^ of soil (Fig. 3). Many samples from 120 to 150 and 150 to 180 cm had values lower than the lowest point of the curve, indicating that they had <10^6^ 16S rRNA gene copies g^−1^ of soil, and inclusion of these samples would have resulted in even lower average abundances in these two lowest soil depths (Fig. 3).

### Effect of crop type and sampling location on the microbial community composition at different soil depths.

**FIG 4:**
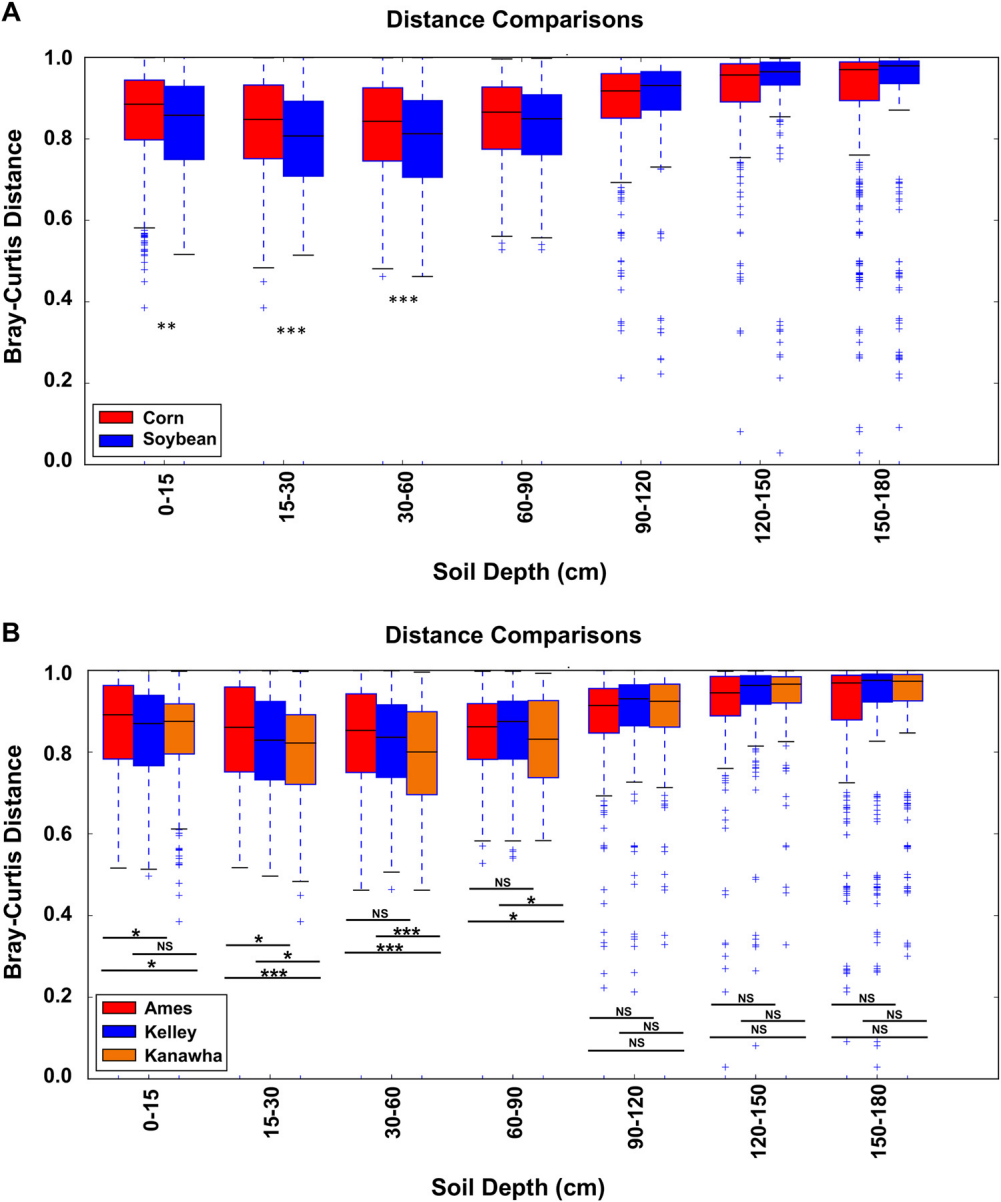
Distribution of pairwise Bray-Curtis dissimilarities between crop type and site at different soil depths. Bray-Curtis distances between soils from corn and soybean fields (A) and between soils (B) from each of three locations along a soil depth gradient were computed using the “make_distance_comparison_plots.py” function in QIIME 1. Significance tests were performed using two-sided Student’s two sample *t* test. Asterisks indicate significant differences (**, 0.01; ***, 0.001). NS, not significant.

To assess if crop type or sampling location influenced microbial community composition at different soil depths, a pairwise comparison of Bray-Curtis dissimilarities was used between the soils from corn and soybean fields (Fig. 4A) or among the three sampling locations, including Ames, Kelley, and Kanawha (Fig. 4B), along the soil depth gradient. Soil microbial community composition was significantly different between the two crop types at the top three depths, including 0 to 15 cm (*P ≤ *0.01), 15 to 30 cm (*P ≤ *0.001), and 30 to 60 cm (*P ≤ *0.001), and were not different from each other at soil depths deeper than 60 cm (Fig. 4A). More variation of soil microbial community composition was observed across the three sampling locations up to a soil depth of 90 cm, while no significant variation was observed in deeper soil layers. The soil microbial community of Kanawha was consistently different from that of Ames at soil depths of 0 to 90 cm and different from Kelley at depths of 15 to 90 cm (Fig. 4B). Ames and Kelley had distinct microbial communities only in the 0- to 30-cm region. These results indicated that both crop type and sampling location had significant effects on the microbial community composition in the upper profile of the soil.

Crop types and locations also affected specific microbial taxa. The three locations showed changes in relative abundance of nine phyla in the surface soil (0 to 15 cm), 10 phyla in the 15- to 30-cm layer, 5 phyla in the 30- to 60-cm layer, and 1 phylum in the 60- to 90-cm layer. Below this depth, there were no differences in phylum relative abundance between locations (Fig. S5). Soybean and corn showed fewer changes at the phylum level when comparing different locations, but changes were observed except in the deepest layers. Four phyla were different between the two crops in the topsoil (0 to 15 cm), one phylum in the 15- to 30-cm layer, two phyla in the 30- to 60-cm layer, no phylum in the 60- to 90-cm layer, six phyla in the 90- to 120-cm layer, five phyla in the 120- to 150-cm layer, and three phyla in the 150- to 180-cm layer. Several genera changed in relative abundance in the surface layers between crops, while only a few differed in the lowest depths (Fig. S6).

### Soil properties correlated with microbial community composition and abundance.

**FIG 5:**
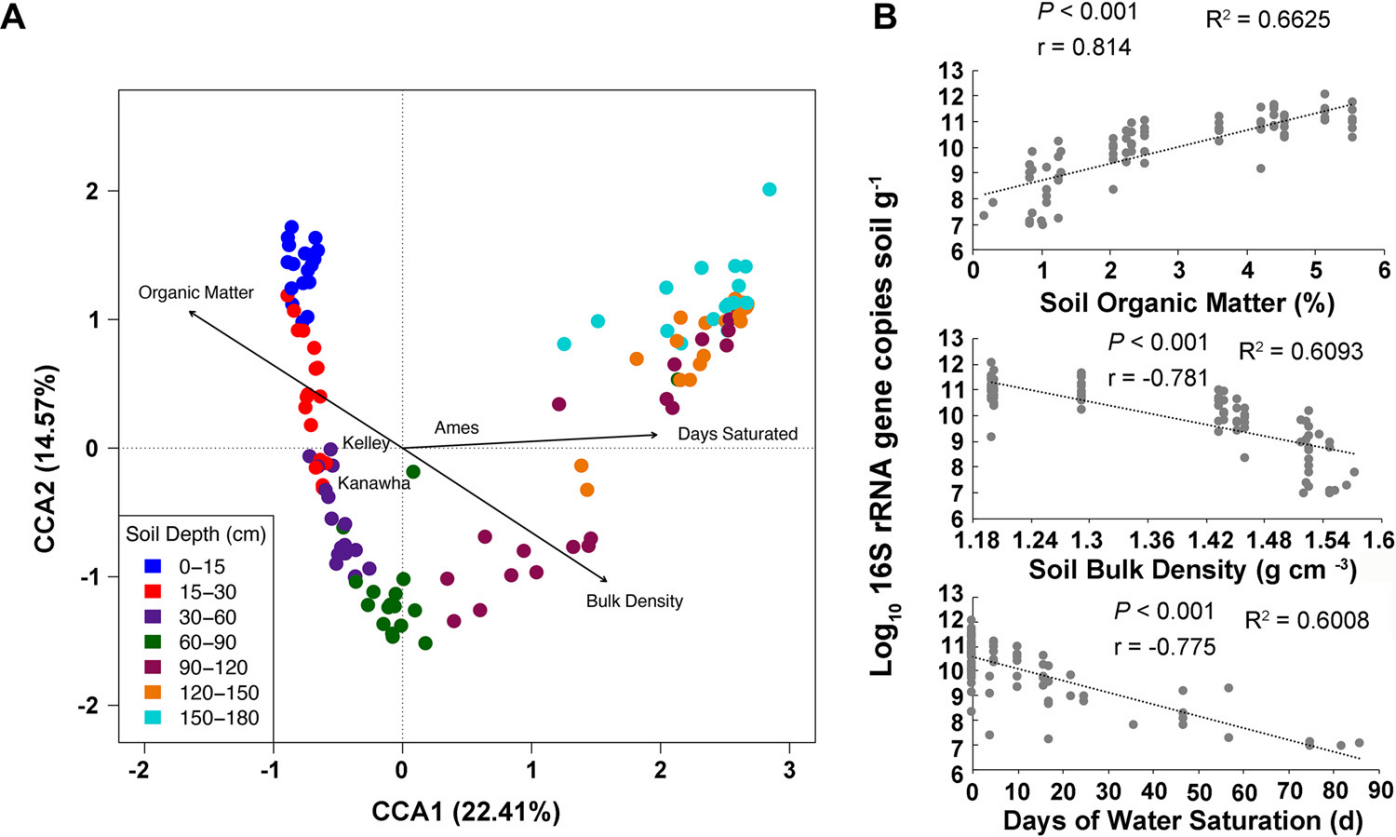
Canonical correspondence analysis (CCA) and correlations of microbial abundance with additional factors influencing soil microbial community composition. (A) CCA1 is the constrained ordination of the data with 22.41% (*P < *0.001) of the variation and CCA2 with 14.57% (*P < *0.001) of the total variation. The significance for each soil property is presented in Table 1. (B) Linear correlation analyses between 16S rRNA gene copies and single soil attributes. The Pearson correlation coefficient and *P* value are shown for each graph.

Canonical correspondence analysis (CCA) showed that soil depth was the most dominant (*P ≤ *0.001) factor shaping the microbial community composition, explaining 15.4% of the variation in microbial communities (Fig. 5A). Sampling site was also significant in this analysis (*P ≤ *0.001), explaining 3.1% of the variation. Among the soil properties analyzed, soil organic matter (*P ≤ *0.001), bulk density (*P ≤ *0.05), and the length of time the subsurface soil was inundated by water (days saturated) (*P ≤ *0.001) were significant in explaining the variation in soil microbial community composition. The soil organic matter accounted for 2.1% of the total variation in microbial community, and length of time the subsurface soil was inundated by water explained 1.5% (Table 1). Other variables, including root biomass, root length, and plant water availability, were not statistically significant (*P > *0.05) in influencing the soil microbial communities. Three properties were also significantly correlated (*P < *0.001) with microbial abundance (16S rRNA gene copies) (Fig. 5B). Soil organic matter was the variable explaining most of the variation (*R*^2^ = 0.662) and was most correlated (*r* = 0.814) with changes in microbial abundance along the soil profile (Fig. 5B). Soil bulk density and days of water saturation were both negatively correlated with microbial abundance (*r* = −0.781 and −0.775, respectively).

**TABLE 1:** CCA of changes in microbial community composition due to soil properties and certain plant parameters^*a*^

Parameter	Df	Chi-square	*F*	*P* (>*F*)	Significance
Depth	6	1.9562	3.8015	0.001	***
Site	2	0.3876	2.2598	0.001	***
Days saturated	1	0.2003	2.3349	0.001	***
Organic matter	1	0.1558	1.8165	0.001	***
Bulk density	1	0.1425	1.6610	0.002	**
Crop	1	0.1033	1.2043	0.086	
Root length	1	0.1249	1.4558	0.075	
Root biomass	1	0.0819	0.9545	0.451	
Plant-available water	1	0.1105	1.2887	0.106	
Residual	111	9.4342			

a Model: cca (formula = d2 ∼ depth + site + days_saturated + organic_matter + bulk_density + root_length + root_biomass + plant_available_water + crop, data = d1, na.action = na.exclude, scale = TRUE, center = TRUE). Asterisks indicate significant differences as follows: ***, *P* = 0.001; **, *P* < 0.01. Df, degrees of freedom; *P*(>*F*), *P* value of the ANOVA test.

An additional CCA using preliminary data comprising single unreplicated values of pH at each depth for each field indicated that pH was a significant factor shaping the microbial community composition. Since data in each field were unreplicated, we did not include these results in our study, but pH is potentially important in structuring microbial communities along a depth profile as previously shown for surface soils (37).

### Changes in specific microbial taxa along soil depth.

**FIG 6:**
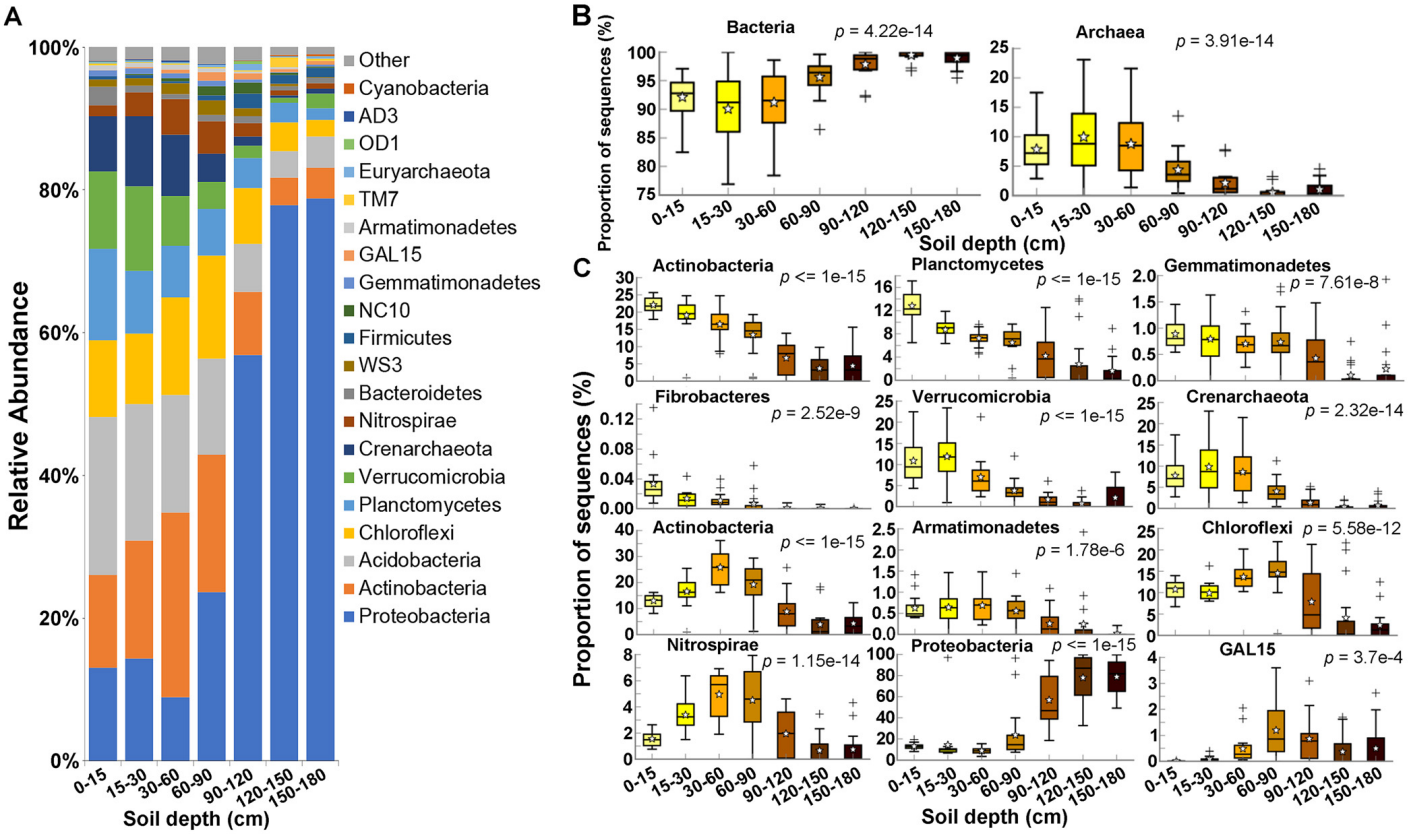
Relative abundances of the dominant microbial phyla in all samples separated by soil depth. (A) Phylum-level relative abundance of the top 20 most abundant taxa. (B and C) Statistical comparison of each domain (B) and phylum (C) relative abundance at each soil depth using Welch’s *t* test and the Bonferroni *P* value correction.

A total of 31,230 ASVs were identified and assigned to 53 phyla, 138 classes, 208 orders, 238 families, and 306 genera for all 126 soil samples. The dominant microbial phyla across all samples included *Proteobacteria*, *Actinobacteria*, *Acidobacteria*, *Chloroflexi*, *Planctomycetes*, *Verrucomicrobia*, *Crenarchaeota*, *Nitrospirae*, which accounted for more than 90% of bacterial and archaeal sequence reads (Fig. 6A). The relative abundance of *Acidobacteria* gradually declined with depth, whereas *Actinobacteria* increased in relative abundance from 0 to 60 cm and decreased gradually with depth in the deeper horizons (60 to 180 cm). The phyla *Verrucomicrobia* and *Crenarchaeota* were relatively more abundant in the surface soil layers (0 to 60 cm) than in the deeper regions (60 to 180 cm), although they accounted for a relatively small proportion of the microbial community in all soil layers (Fig. 6A). Additional information regarding specific ASVs in the deep soils can be found in Table S2.The statistical results at the domain level showed that the bacterial and archaeal relative abundances were changed significantly along the soil profile (Fig. 6B). The microbial community was dominated by *Bacteria* (>90% average relative abundance) in all depths. However, the abundance of *Archaea* increased proportionally from 15 to 60 cm compared to that in the 0- to 15-cm layer. Below 60 cm, the archaeal relative abundance decreased dramatically (<5% of average relative abundance) and bacterial sequences were almost exclusive in the lowest depths (Fig. 6B).

Twenty phyla showed significant differences in relative abundance (Table S3). Among those showing higher *P* values and effect sizes (η^2^), *Acidobacteria*, *Planctomycetes*, *Gemmatimonadetes*, and *Fibrobacteres* showed a consistent decrease, while *Proteobacteria* showed a consistent increase in relative abundance with depth (Fig. 6C). The increase in *Proteobacteria* with depth was mainly caused by the *Gammaproteobacteria* class (*P < *0.001; η^2^ = 0.601), though a subtle increase in *Betaproteobacteria* was also observed (*P = *0.029; η^2^ = 0.198). On the other hand, some phyla showed an initial increase in relative abundance from the surface to the subsurface soils and then decreased at greater depths: *Verrucomicrobia* and the archaeal phylum *Crenarchaeota* showed maximum relative abundance at 15 to 30 cm, *Actinobacteria* and *Armatimonadetes* showed maximum relative abundance at 30 to 60 cm, and *Chloroflexi* and *Nitrospirae* showed maximum relative abundance at 60 to 90 cm. The two lowest depths were dominated by *Proteobacteria* (Fig. 6C).

STAMP analysis identified the described/known genera changing in relative abundance along soil depth. Fifty-eight genera were significantly different along the soil profile using this approach; most of them decreased in relative abundance from topsoil to subsoil (Table S4). The main 15 genera (higher *P* value and effect size) changing in relative abundance are shown in Fig. S8. Among them, only three increased in relative abundance with depth, i.e., *Escherichia*, *Hyphomicrobium*, and *Phyllobacterium* (Fig. S8A). However, *Hyphomicrobium* and *Phyllobacterium* showed a decrease after 90 cm, while the microbial community was dominated by *Escherichia* from 90 to 180 cm. On the other hand, the most significant genera decreasing in relative abundance with depth were *Pseudonocardia*, *Microlunatus*, *Flavisolibacter*, *Pilimelia*, *Nonomuraea*, *Actinoplanes*, *Sorangium*, *Pirellula*, *Geodermatophilus*, *Prosthecobacter*, *Streptosporangium*, and *Pedomicrobium* (Fig. S8B).

Consistent with the spatial distributions of the microbial phyla along the soil depth gradient, analysis of composition of microbiomes (ANCOM) revealed that the genus *Escherichia* of the phylum *Proteobacteria* drastically increased in abundance starting from soil depth below 60 cm (Fig. S7). In contrast, the orders RB41 and WD2101 of the phyla *Acidobacteria* and *Planctomycetes*, respectively, showed a decrease in abundance with soil depth. The genera *DA101* and “*Candidatus* Nitrososphaera,” of the phyla *Verrucomicrobia* and *Crenarchaeota*, respectively, were in greater abundance in the soil depth of 15 to 30 cm. The genus JG37-AG-70 and the orders SB-34 and 0319-7L14, of the phyla *Nitrospirae*, *Chloroflexi*, and *Actinobacteria*, respectively, were more abundant at the soil depth of 30 to 90 cm (Fig. S7). Additional information about the taxonomy groups that exhibited differential abundance across soil depths can be found in Table S5.

## DISCUSSION

This study demonstrated that the composition and diversity of microbial communities in highly productive soybean and corn fields (32) were strongly impacted by soil depth. Some of the major factors involved in shaping community profiles included various edaphic factors, such as soil organic matter, bulk density, and water retention. These findings are consistent with previous studies showing that soil depth is a fundamental environmental factor shaping the soil microbiome (38). The samples from agricultural fields used in our study provided strong evidence that the effect of soil depth on structuring microbial communities is very important in agroecosystems. Few studies on the impact of soil depth in agricultural soils are found in the literature (10, 20, 39, 40), since the majority of past studies focused on nonagricultural soils (11, 15, 18, 19, 22, 23, 38, 41–44).

Among the edaphic factors explaining the microbiome variation along the soil profile, bulk density was correlated with the microbiomes inhabiting increased soil depths (mainly 90 to 120 cm). The higher soil compaction in the deeper layers reduces O_2_ availability (45, 46), limiting the growth of many microbial taxa (47). Higher soil bulk density also tends to increase water retention (48), which may explain why the microbiomes from the deepest soil layers in our study (120 to 180 cm) were correlated with days of water saturation (49). Correlation of microbiome composition with soil organic matter (SOM) in the surface soil layers (0 to 30 cm) may be due to the greater accumulation of labile organic compounds in the topsoil, which has been shown to shape the heterotrophic microbiota (50, 51). There is evidence that deep soils also store large amounts of carbon (52, 53), but how the carbon interacts with microbes was not studied in this investigation. Although root length and biomass were not significant in structuring the microbial communities along the soil profile in our study, deep roots may be one source of carbon deposition in subsurface soil layers and thus may indirectly affect the microbiome in deep soils (52, 54, 55). We also showed a decrease in microbial alpha diversity and an exponential decrease in bacterial abundance from surface to subsurface soils; such decreases were previously detected in forests and some agricultural soils, highlighting the more restrictive conditions for microbial life in deeper soils (11, 22, 38, 40, 42, 43, 56, 57). The variation from surface soil to deep soils in forests from Brazil and New Zealand were consistent with the abundance observed in our study (in general 10^11^ to 10^6^ copies g^−1^ of soil) (42, 58). In one study, reduced carbon availability was suggested as a main reason for decreased microbial biomass in deeper soil layers (11). Our study confirmed that SOM content is highly correlated with bacterial abundance along soil depth. In addition to impacting the alpha and beta diversity of microbial communities, soil bulk density and water saturation were also important in determining bacterial abundance along the soil profile, as soil microbes are mainly aerobic and therefore inhibited by low oxygen availability (47, 59).

A previous study assessing changes in microbial communities along a soil depth profile in wheat fields detected a proportional decrease in *Archaea* with soil depth (39). In contrast, our data showed an initial increase in archaeal relative abundance from 15 to 60 cm and then a dramatic drop after 60 cm. The increase in archaeal relative abundance to a depth of 60 cm was also observed in *Crenarchaeota*, which play a pivotal role in soil nitrification through ammonia oxidation (50, 60). Since carbon availability is usually lower in deep soils, a decrease in relative abundance of heterotrophic microbes would be expected along with an increase in chemolithoautotrophs, such as the ammonia-oxidizing archaea (50, 60).

The relative abundance of many microbial phyla changed along the soil profile in our study. The decrease in relative abundance of *Acidobacteria*, *Verrucomicrobia*, *Gemmatimonadetes*, and *Planctomycetes* with depth was also observed in previous studies (11, 15, 19, 20, 38, 43, 61). *Gemmatimonadetes* is usually considered copiotrophic, which may be one reason why it decreased with depth, since carbon availability is lower in deeper layers (62). *Planctomycetes* is primarily considered oligotrophic (62), but the large genome size of these bacteria suggests a more copiotrophic lifestyle, which would favor their growth in the upper regions of the soil profile (63). *Acidobacteria* is considered to be oligotrophic (62), but this phylum is often associated with changes in soil pH and its abundance generally increases in more acidic soils (64). The lower relative abundance of *Acidobacteria* in deeper soils may have been associated with the increased soil pH (38) with depth. Our study detected a slight increase in *Verrucomicrobia* relative abundance from 15 to 30 cm, but this oligotrophic phylum decreased thereafter, as shown in other studies (20, 38, 43). These data support the idea that factors such as lifestyle and specific edaphic characteristics may play a major role in the adaption of bacteria and archaea to life at different soil depths.

Representative taxa from the phyla *Actinobacteria*, *Nitrospirae*, and *Chloroflexi* progressively increased in relative abundance from 15 to 90 cm. This increase with depth in these phyla was also detected in previous studies (10, 15, 18–20, 22, 23, 38, 40, 41). Despite being considered copiotrophic, *Actinobacteria* has the ability to degrade recalcitrant carbon sources and is potentially favored in deeper soils compared to other microbes due to decreased amounts of labile carbon at lower depths (62). There are many potential reasons that *Chloroflexi* may have been enriched in deeper soils. It is considered to be oligotrophic, and many strains in this phylum are anaerobic and chemolithotrophs or able to respire organohalides, which are advantageous attributes for growth under the low-redox-potential conditions of deeper soils (65–67). Similarly, the oligotrophic phylum *Nitrospirae* is also potentially favored under those conditions, since there are aerobic and anaerobic chemolithoautotrophic strains with key roles in soil nitrification (68).

Consistent with a study on a wide range of soil ecosystems, including grasslands, forest, and prairies (69), we found that DA101 was one of the most abundant genera in the topsoil of agricultural land. This enrichment of DA101 in the topsoil is probably due to the elevated carbon released by plant roots (69). Below 90 cm the soil microbial community was dominated by *Proteobacteria*, a phenomenon also observed in other studies (39, 57). In our study, the dominance of *Proteobacteria* in the deepest soil layers was primarily determined by the genus *Escherichia*. Although best known as enteric bacteria, *Escherichia* spp. can persist for a long time in soils and have many characteristics that may have caused dominance in the deepest layers, such as an ability to obtain a diversity of nutrients from the environment, tolerate stress conditions, and remain viable for long periods in a dormant state (70–74). Data suggest that the presence of *Escherichia* was not due to contamination because the abundance of this ASV was 10 to 100 times higher than in blank controls (no soil DNA) used in the sequencing. Whether the dominance of *Escherichia* spp. in the deepest profile was caused by very strong selective pressure (low oxygen levels, redox potential, and carbon availability) or by ecological drift due to a random establishment of isolates in a habitat with reduced bacterial abundance is an open question to be researched in future studies (75).

Other proteobacterial genera also increased in relative abundance to a depth of 120 cm, including *Hyphomicrobium* and *Phyllobacterium*, both from the *Rhizobiales* order. *Hyphomicrobium* is a methylotrophic genus of bacteria known to act synergistically with methanotrophic bacteria, which are probably favored in deeper soils, since they often have a higher moisture content and lower redox potential, probably leading to enhanced methanogenesis (76, 77). *Phyllobacterium* comprises bacteria that are able to associate with roots of many plants and perform N fixation (78). There are no reports of *Phyllobacterium* spp. associated with maize or soybean roots, and therefore, the enrichment of this genus in the subsurface may be unusual.

The location of each field was the second most important factor that impacted soil microbial communities in our study after soil depth. However, the influence of sampling site location on soil microbial communities was only significant above a depth of 90 cm. Microbial community structure of the Kanawha site was more distinct than those of the Ames and Kelley sites until the deepest layers, which may be due to the geographical separation of Kanawha from Ames and Kelley. Although soil tillage strongly impacts microbial community structure and diversity (79, 80), the microbial communities from Ames (conventional tillage) and Kelley (no-till site) were more similar to each other than to that of Kanawha (conventional tillage). As bacterial biogeography has been shown to be primarily controlled by edaphic factors rather than geographical distance (37), the soil physicochemical differences between these distant locations are likely to be a main reason for the distinct nature of Kanawha soils compared to the other sites. In addition, soil moisture plays important roles in shaping microbial communities (81). In the year these samples were collected, there was a larger amount of precipitation in Kanawha during the growing season, while the average temperatures were lower than in Ames and Kelley, which could also contribute to a larger difference in the microbiome of the Kanawha topsoil than for the other two sites.

In addition to depth and site, crop type also contributed significantly to the variation in microbial communities, but only at the upper regions of the soil profile. These results are consistent with a previous study investigating the effects of different crops on soil communities at different soil depths from 0 to 100 cm (82, 83). In the present study, soybean and corn root biomass was greater in the top 90 cm, while differences were observed in microbial composition in the first 60 cm. These upper regions, where more roots are found, would be richer in plant root exudates (83–85), which may account for the differences in soil microbial composition between crops. In addition to exudates, plant residues (root and shoot litter) that differ in C:N ratio, as is the case for maize compared to soybean (86, 87), may also affect soil microbial communities. The effect of crop type on soil microbial communities was not detected at deeper soil depths, potentially due to the small amount of root biomass and plant residues in deeper soils. In a previous study at these sampling sites, no significant difference in root biomass was found between soybean and corn crops below 90 cm of soil depth at each site (*P* > 0.22) (88).

### Conclusion.

Soil depth is a fundamental factor in structuring soil microbial communities in agricultural soils, and deep soils are a critical zone for soil formation and carbon sequestration. Decreased bacterial abundance, species richness, and diversity were observed in deep compared to surface soils. Field site and crop type significantly contributed to the variation in microbial communities only in the upper soil layers. Among the measured soil properties, soil organic matter, soil bulk density, and the time that deep soils were saturated with water were significant factors explaining the variation in soil microbial community composition. Distinct distribution patterns in microbial community composition along soil profiles were measured, with *Proteobacteria* dominating the deeper soils. The development of a better understanding of changes and factors that influence plant-microbe-soil interactions through the soil profile in the agroecosystems should enable more strategic deployment of plant and microbial solutions to improve crop yields and to mitigate the adverse environmental effects of agriculture while enhancing food production to feed burgeoning world populations.

## MATERIALS AND METHODS

### Field sites and soil sample information.

Soil samples were collected from corn and soybean fields at Ames, Kelley, and Kanawha, located near Des Moines IA. Soybean (Glycine max) and corn (Zea mays) were planted in Ames and Kelley, while corn was the only crop in Kanawha. The Kelley site had subsurface tile drainage installed at 1.1 m below the surface, so the 0- to 1-m soil profile rarely was saturated with water. In contrast, Ames and Kanawha, which had the same soil type as Kelley (Nicollet soil series), had no tile drainage, so the 0- to 1-m profile was saturated with water for longer periods. Recent experimental and modeling studies carried out in these fields showed that the depth of the water table and the hydrology of the field dictate the corn and soybean root distribution (88–90). The Kelley site has been under no-till management since 2009; the other two sites were tilled every autumn. Corn plots were fertilized with urea at rates of 134, 168, and 336 kg of N ha^−1^ at Kelley, Ames, and Kanawha, respectively. Fertilizer was broadcast at preplanting, while plots cultivated with soybeans received no added N (89).

Deep soil cores were collected in the plant row during the mid-grain filling period (a period when root mass is maximum) (91) using a 6.2-cm-diameter Giddings probe. One soil core was collected in each of three plots that were arranged in randomized block design. The deep cores were sectioned into seven depth intervals (0 to 15, 15 to 30, 30 to 60, 60 to 90, 90 to 120, 120 to 150, and 150 to 180 cm). A total of 126 samples from corn and soybean fields at the three sampling sites (Ames, Kelley, and Kanawha) were included in this study. Soil samples were packaged in Ziploc plastic bags and kept in a cooler with ice packs. Soil samples were transported to the lab, and each soil layer sample (about 2 kg) was passed through a sieve of 530 mm to break up soil aggregates and mixed properly. A single tube containing 5 ml of soil was sampled from soil for microbial analysis. Across the field trials, the samples were collected in July 2017 for maize and August 2017 for soybean.

The remaining soil samples were processed to determine root properties, the detail of which were previously published (92). Soil samples were soaked in 10 g liter^−1^ of sodium hexametaphosphate solution to break up soil aggregates and sprayed with pressurized water to float the roots, which were recovered using a 530-µm sieve. Root tissue was oven dried, and root dry weight was determined. Soil textural data were measured on in-row cores from each plot using laser diffractometry (93) with a Malvern Mastersizer 3000 and a HydroEV attachment (Malvern Panalytical Ltd., UK) on 30-cm-soil-depth increments. Soil carbon (C) was measured at each depth increment at each site. Pedotransfer functions utilizing soil texture and soil C measurements were used to calculate bulk density and plant-available water for each soil layer using the appropriate equations (32, 94). Soil and root properties at the three experimental sites are listed in Table S1.

### DNA extractions, 16S rRNA gene amplification, and sequencing.

DNA was extracted from soil samples using the PowerSoil-htp 96-well soil DNA isolation kit (MoBio, Carlsbad, CA). The V4 region of the 16S rRNA gene was amplified by PCR using a dual-index sequencing strategy (95) with AccuPrime *Pfx* DNA polymerase (Invitrogen, Carlsbad, CA). A dual-index primer system was used and consists of the Illumina adapter, an 8-nucleotide index sequence, a 10-nucleotide pad sequence, a 2-nucleotide linker sequence, and the 16S rRNA V4 primer (95). Amplification reactions were checked by running PCR products on a 1% agarose gel to ensure success of the PCR. The PCRs were purified and normalized using SequalPrep normalization plates (Invitrogen). The concentration of PCR products was measured using the QuantiFluor double-stranded DNA (dsDNA) system (Promega, Madison, WI) and used to pool equimolar amounts of PCR products. Pooled samples were concentrated using a SpeedVac, and fragments within a size range of 200 to 700 bp were size selected using the SPRIselect beads (Beckman Coulter, Brea, CA). In the amplicon library, a blank DNA extraction control was used as a negative control. Genomic DNA from microbial mock community B (even, low concentration), v5.1L 16S rRNA gene sequencing (BEI Resources, Manassas, VA), was also amplified and included in each sequencing run. Sequencing libraries were quantified and quality checked using a high-sensitivity DNA kit on an Agilent 2100 Bioanalyzer (Agilent Technologies, Santa Clara, CA). Sequencing was performed on the Illumina MiSeq platform using the MiSeq reagent kit v3 (600 cycles; Illumina, San Diego, CA) with a spiking of 20% PhiX control library (Illumina).

### Quantification of 16S rRNA gene copies.

Bacterial abundance along soil depth was estimated by quantifying the number of 16S rRNA gene copies using quantitative real-time PCR. Amplifications were performed using a 10 µM concentration of the primers 341F (5′-CCTACGGGAGGCAGCAG-3′) and 534R (5′-ATTACCGCGGCTGCTGG-3′) targeting the V3 region of the bacterial 16S rRNA gene (96), 12.5 µl (2×) of Power SYBR green PCR master mix (Applied Biosystems, Carlsbad, CA) and 1 µl of template DNA for a total volume of 25 µl (97). PCR was conducted in a CFX Connect real-time system (Bio-Rad, USA) under the following thermal cycling conditions: initial denaturing at 95°C for 3 min and 35 cycles of 94°C for 30 s, 55°C for 30 s, and 72°C for 30 s, followed by a melting-curve analysis. For the standard curve, soil-derived amplicons (using the same primer pair) were serial diluted from 10^−1^ to 10^−9^ and quantified (58, 98). The standard curve was subjected to amplifications using the same conditions as described above, as well as negative controls. The samples with higher values than the lowest concentration of the standard curve (10^−9^) were used to quantify bacterial abundance. The *R*^2^ values of the standard curves in all plates were higher than 0.99, and the PCR efficiency ranged from 100.83 to 105.17%, between the accepted values of 90 to 110%, indicating the absence of PCR inhibitors.

### Sequence processing.

The raw paired-end sequencing reads were processed using USEARCH (version 10.0.240) and QIIME (Quantitative Insights into Microbial Ecology, version 1.9.1) (99). Briefly, sequence reads were demultiplexed and high-quality merged reads were clustered with simultaneous chimera removal using UNOISE implemented in USEARCH into amplicon sequence variants (ASVs) based on 100% sequence similarity. ASVs were classified using the Ribosomal Database Project (RDP) classifier (100) against the Greengenes 16S rRNA gene database (18). Chloroplast and mitochondrial sequences were identified and removed from the data. Low-abundance ASVs (<2 total counts) were discarded. All samples were rarefied to 5,024 sequence reads per sample, and samples having fewer sequence reads were removed. The microbial alpha diversity was evaluated by calculating the observed ASVs (species richness) and Shannon, Simpson, and Faith’s diversity indices (species diversity).

### Statistical analyses.

The microbial beta diversity was assessed by calculating the Bray-Curtis dissimilarity between samples. Canonical analysis of principal coordinates (CAP) was conducted using the “capscale” function in the vegan (v2.5.3) R package (101). Data visualization was performed using ggplot2 (v2.2.1) (102). The taxon shift along with different depths is presented in bar plots based on the percent relative abundances of the top 20 most abundant microbes at the phylum level. Analysis of composition of microbiomes (ANCOM) was performed to identify taxonomy groups that were differentially enriched at different soil depths (103). In addition, changes in relative abundance at specific taxonomic levels (i.e., domain, phylum, and genus) were assessed using Welch’s *t* test with Bonferroni *P* value correction in STAMP software (104).

Canonical correspondence analysis (CCA) was conducted to explore the relationship between the microbial community composition and soil properties (soil physicochemical variables listed in Table S1) which were reported in an earlier study (32) using the “cca” function in R (101). Soil properties that led to statistically significant changes in microbial community composition were selected to build the CCA model using the “ordistep” function with 999 permutations. Statistical significance of each soil property and CCA axes were determined using the Monte Carlo permutation test with 999 permutations.

Differences in microbial alpha diversity were determined using the Wilcoxon test adjustment for false-discovery rate implemented in R. Permutational multivariate analysis of variance (PERMANOVA) was performed to assess the effects of soil depth, sites, and crop type on microbial community data using the “adonis” function in the vegan R package (101). Pairwise comparisons of Bray-Curtis dissimilarities between corn and soybean soil across soil depth were conducted using two-sided Student’s two-sample *t* test. A *P *value of <0.05 was considered statistically significant. Comparison of 16S rRNA gene copy numbers between soil layers was performed using ANOVA and Tukey’s pairwise test. In addition, linear regressions and linear correlation (Pearson) analyses were performed between soil attributes and 16S rRNA gene copies using Past software (105).

### Data availability.

The 16S rRNA sequences used in this study have been submitted to the NCBI Sequence Read Archive (SRA) with BioProject accession number PRJNA638682.

## Supplementary Material

Supplemental file 1

Supplemental file 2

Supplemental file 3

Supplemental file 4

Supplemental file 5

Supplemental file 6
