# Supplementary material for: The Effects of Soil Depth on the Structure of Microbial Communities in Agricultural Soils in Iowa (United States)
Source: Appl Environ Microbiol. 2021 Jan 29;87(4):e02673-20. doi: 10.1128/AEM.02673-20 (PMC7851703; doi:10.1128/AEM.02673-20)
Supplement: Supplemental file 6 [file AEM.02673-20-s0006.pdf]

Fig. S1. Alpha diversity indices at different sampling sites at each soil depth. (A) Average number of observed ASVs, (B) Shannon index, (C) Simpson index and (D) Faith's phylogenetic diversity index at different sites along soil depth. Differences in alpha diversity were compared using Wilcoxon test adjust for false discovery rate. $P < 0.05$ was considered statistically significant. Line in the box represents median. The top and bottom of box represent the first and the third quartile, respectively. Whiskers indicate data's range with outliers showing as black dots. All the pairwise comparisons were made and only the ones noted with asterisk were significantly different from another.

Fig. S2. Alpha diversity indices or between different crop types at each soil depth. (A) Average number of observed ASVs, (B) Shannon index, (C) Simpson index and (D) Faith's phylogenetic diversity index between different crop types along soil depth. Differences in alpha diversity were compared using Wilcoxon test adjust for false discovery rate. $P < 0.05$ was considered statistically significant. Line in the box represents median. The top and bottom of box represent the first and the third quartile, respectively. Whiskers indicate data's range with outliers showing as black dots. All the pairwise comparisons were made and there were no significant differences detected between the two crop types.

Fig. S3. Beta diversity showing changes in microbial community composition with depth. Canonical analysis of principal coordinates (CAP) using Bray-Curtis dissimilarity for all samples. The Bray-Curtis dissimilarity matrix was generated using QIIME. CAP analysis was conducted by constraining for only soil depth using the 'capscale' function in vegan R package. PERMANOVA was performed to determine

whether the differences between soil depths was significant with site and crop factored out. Each color indicates a different soil depth as shown in the legend.

Fig. S4. Beta diversity showing changes in microbial community composition with depth. Canonical analysis of principal coordinates (CAP) using (A) weighted UniFrac (WUF) and (B) unweighted UniFrac (UUF) distance metrics for all samples. The WUF and UUF distance metrics were generated using QIIME. CAP analysis was conducted by constraining for soil depth using the 'capscale' function in vegan R package. PERMANOVA was performed to determine whether the differences between soil depths was significant with site and crop factored out. Each color indicates a different soil depth as shown in the legend.

Fig. S5. Changes in phyla relative abundance between the three locations. The phyla with significant ($p < 0.05$) differences between locations based on Welch's t-test are shown for each soil depth layer studied. No significant differences were observed below the 60 - 90 cm layer. The graphs show the specific locations with significant differences for each phylum. The mean proportion of sequences (%) of each phylum, confidence intervals and *p*-values are shown for each comparison.

Fig. S6. Phyla and genera changing in relative abundance between corn and soybean. The phyla and genera showing significant ($p < 0.05$) differences between locations based on Welch's t-test are shown for each soil depth layer studied. No significant difference was observed at the phylum level in the 60 - 90 cm layer. The mean proportion of sequences (%), confidence intervals and *p*-values are shown for each phylum and genus in each soil depth.

Fig. S7. Taxa differentially enriched along soil depth gradient determined by ANCOM. Only the taxa with the highest W-statistic (the higher W-statistics represents the most significant differences in abundance levels) from the most abundant phyla were plotted, including (A) RB41 (B) WD2101 (C) *DA101* (D) *Nitrososphaera* (E) SB-34 (F) *JG37-AG-70* (G) 0319-7L14 and (H) *Escherichia*. The relative abundance of each taxa at different soil depth is shown above the bars.

Fig. S8. The main identifiable genera changing in relative abundance along soil depth. Box plots showing classified genera with significant differences in relative abundance along soil depth according to Welch's t-test with Bonferroni p-value correction. The genera showing the most significant (lower *p*-value) and higher effect sizes (eta-squared) are shown in each panel: (A) genera increasing relative abundance along soil depth and (B) genera decreasing relative abundance along soil depth.

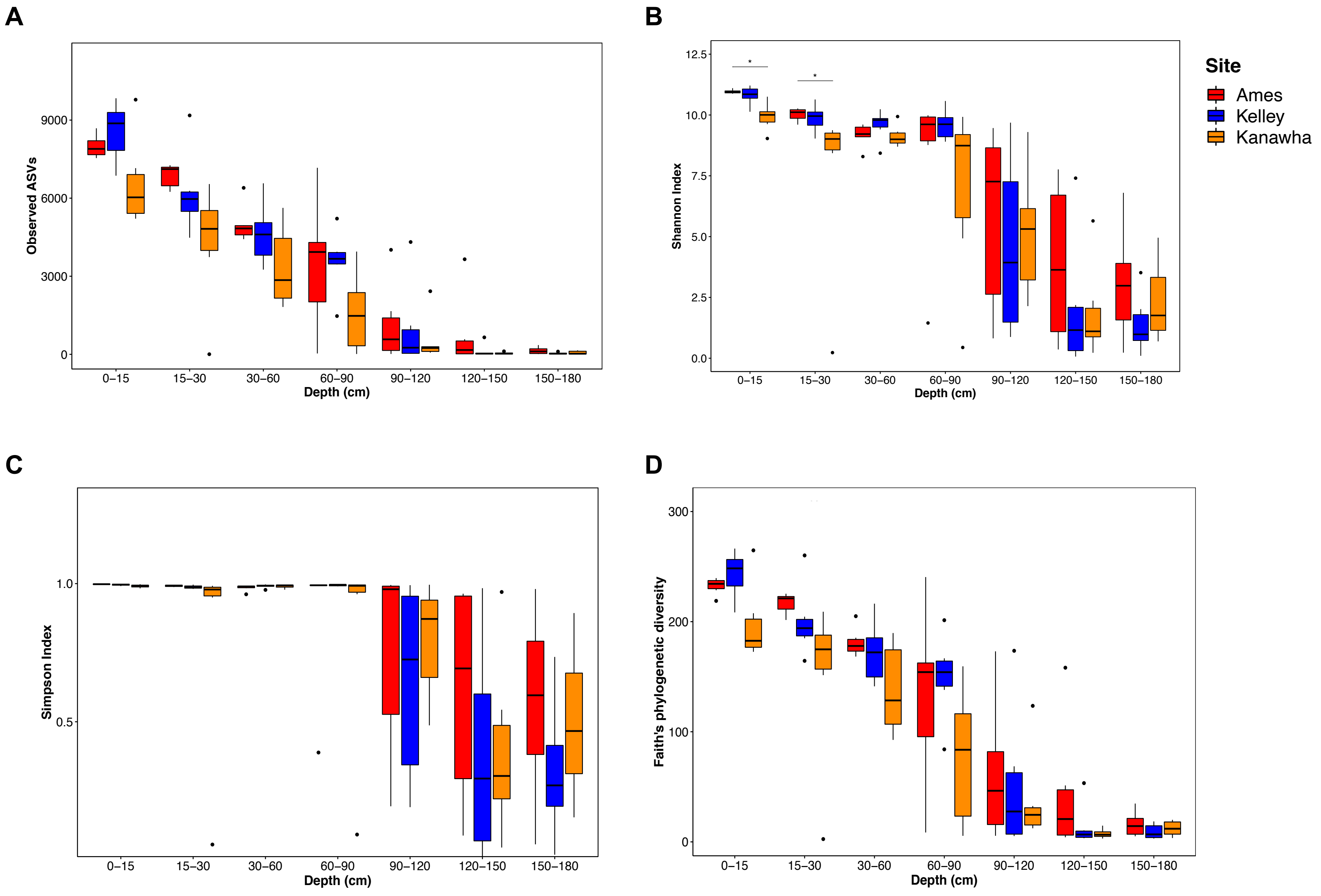
A
B
C
D
Observed ASVs
Shannon Index
Simpson Index
Faith's phylogenetic diversity
Depth (cm)
0–15
15–30
30–60
60–90
90–120
120–150
150–180
Site
Ames
Kelley
Kanawha

Fig. S1

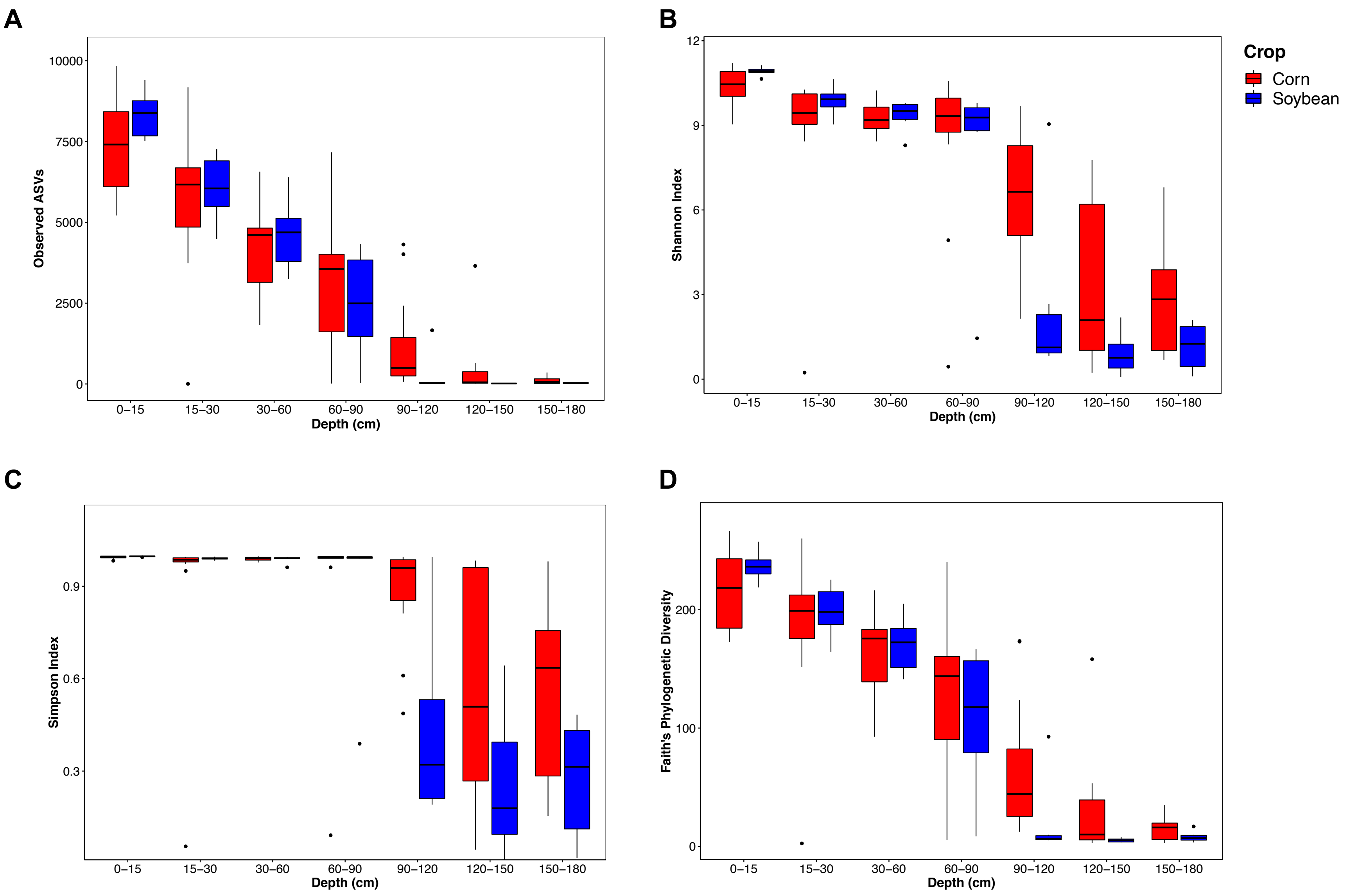
A
Observed ASVs
10000
7500
5000
2500
0
0–15
15–30
30–60
60–90
90–120
120–150
150–180
Depth (cm)
B
Shannon Index
12
9
6
3
0
Crop
Corn
Soybean
C
Simpson Index
0.9
0.6
0.3
D
Faith's Phylogenetic Diversity
200
100
0

Fig. S2

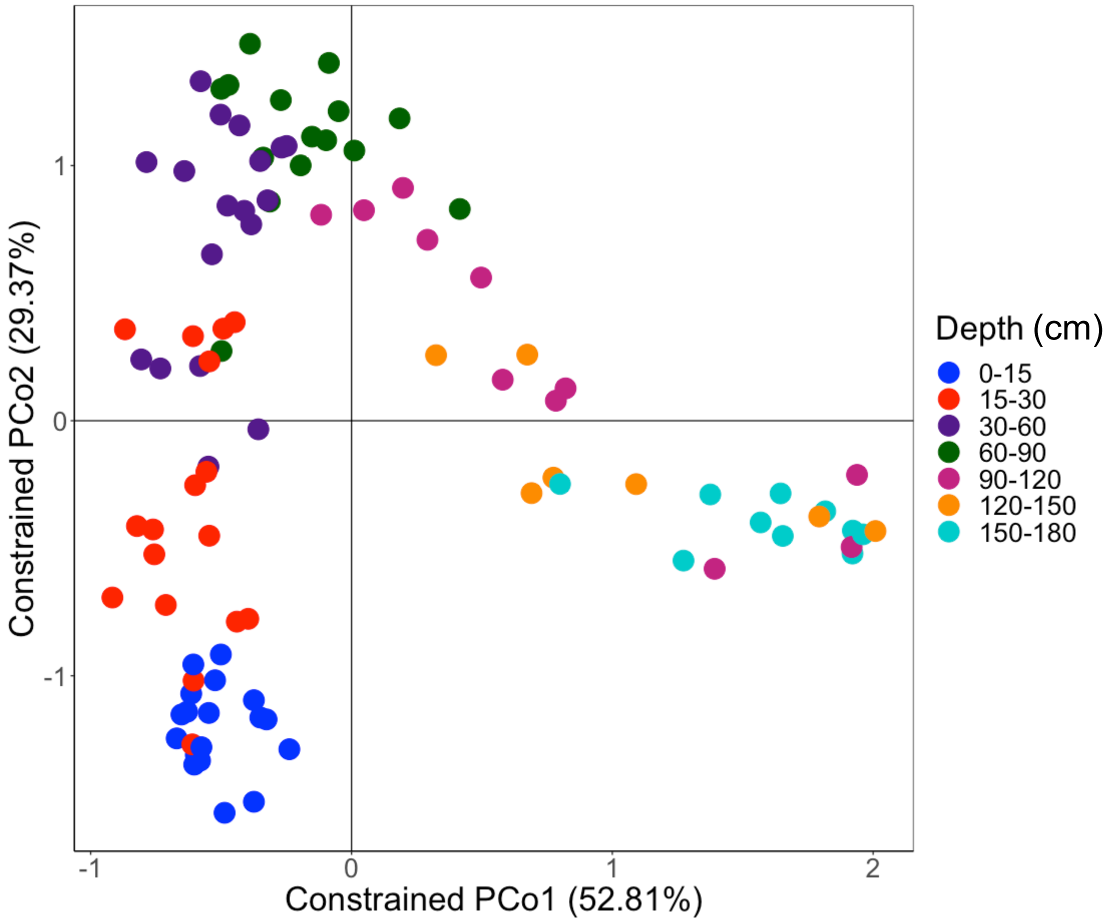

Model: capscale (formula = as.dist(gh.bray) ~ Depth + Condition(Site + Crop), data = gh.map, add = T)

Factor	Df	SumOfSqs	F	Pr(>F)	Signif.
Depth	6	10.965	6.8726	0.001	***
Residual	86	22.867			

Signif. codes: '*' 0.05, '**' 0.01, '***' 0.001

Fig. S3

A

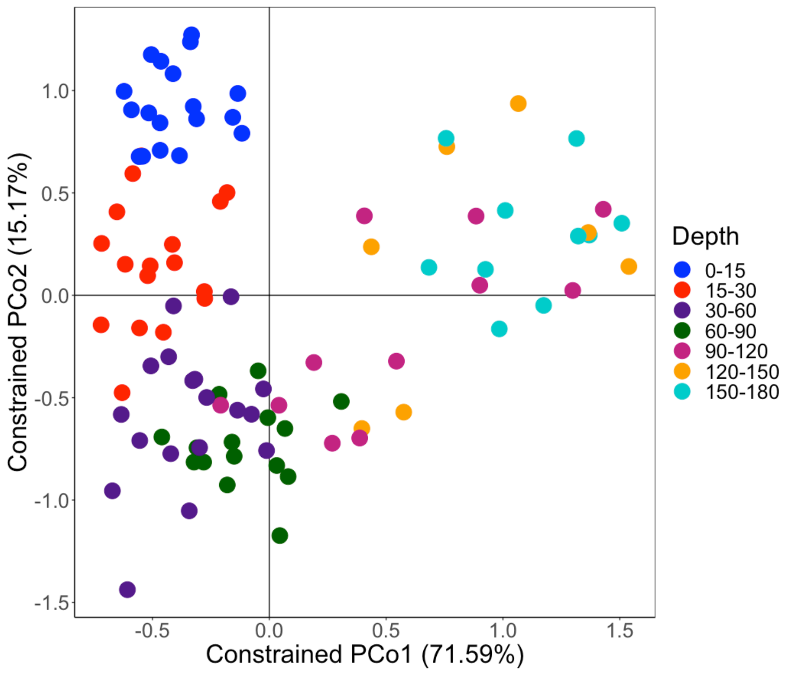

Model: capscale (formula = as.dist(gh.wuf) ~ Depth + Condition(Site + Crop), data = gh.map, add = T)

Factor	Df	SumOfSqs	F	Pr(>F)	Signif.
Depth	6	3.5774	7.766	0.001	***
Residual	86	6.6026			

Signif. codes: '*' 0.05, '**' 0.01, '***' 0.001

B

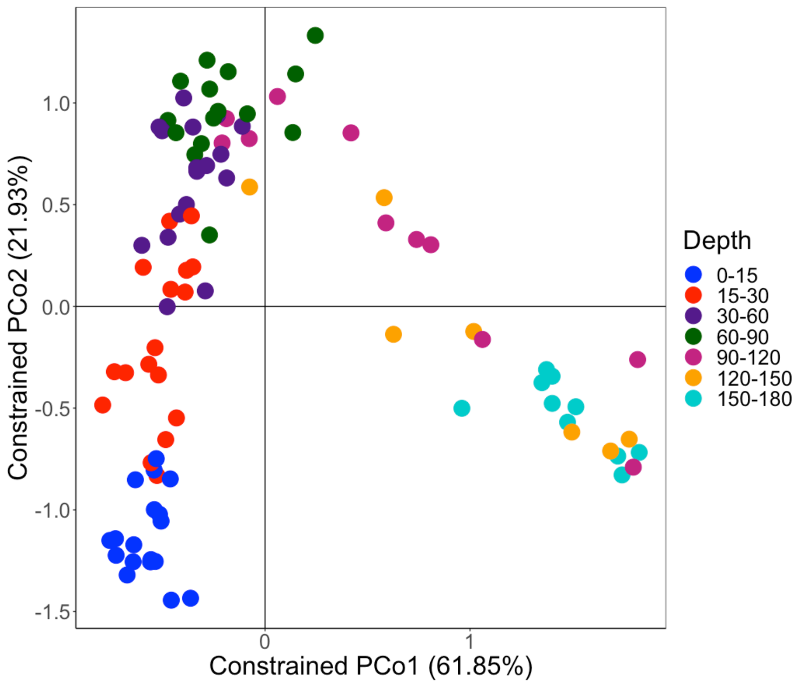

Model: capscale (formula = as.dist(gh.uuf) ~ Depth + Condition(Site + Crop), data = gh.map, add = T)

Factor	Df	SumOfSqs	F	Pr(>F)	Signif.
Depth	6	6.5977	5.4282	0.001	***
Residual	86	17.4212			

Signif. codes: '*' 0.05, '**' 0.01, '***' 0.001

Fig. S4

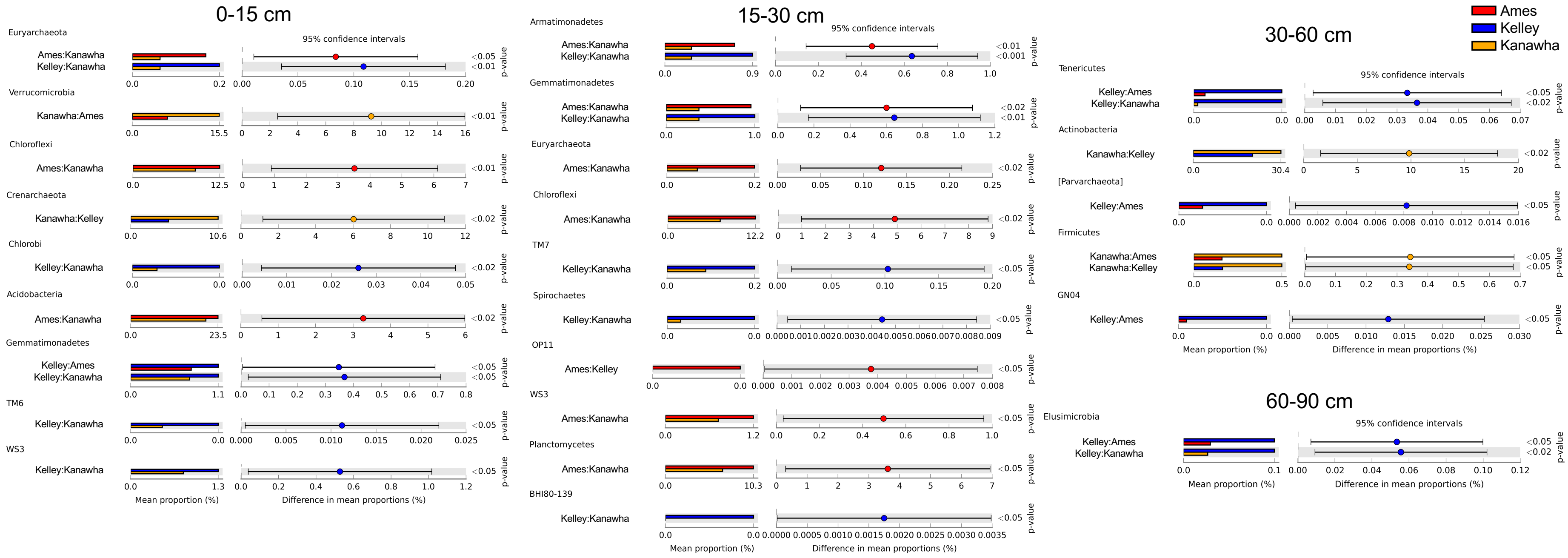

0-15 cm
15-30 cm
30-60 cm
60-90 cm
Ames
Kelley
Kanawha
95% confidence intervals
Euryarchaeota
Ames:Kanawha
Kelley:Kanawha
Verrucomicrobia
Kanawha:Ames
Chloroflexi
Crenarchaeota
Kanawha:Kelley
Chlorobi
Acidobacteria
Gemmatimonadetes
Kelley:Ames
TM6
WS3
Armatimonadetes
TM7
Spirochaetes
OP11
Ames:Kelley
Planctomycetes
BHI80-139
Tenericutes
Actinobacteria
[Parvarchaeota]
Firmicutes
Kanawha:Ames
GN04
Elusimicrobia
Mean proportion (%)
Difference in mean proportions (%)
p-value

Fig. S5

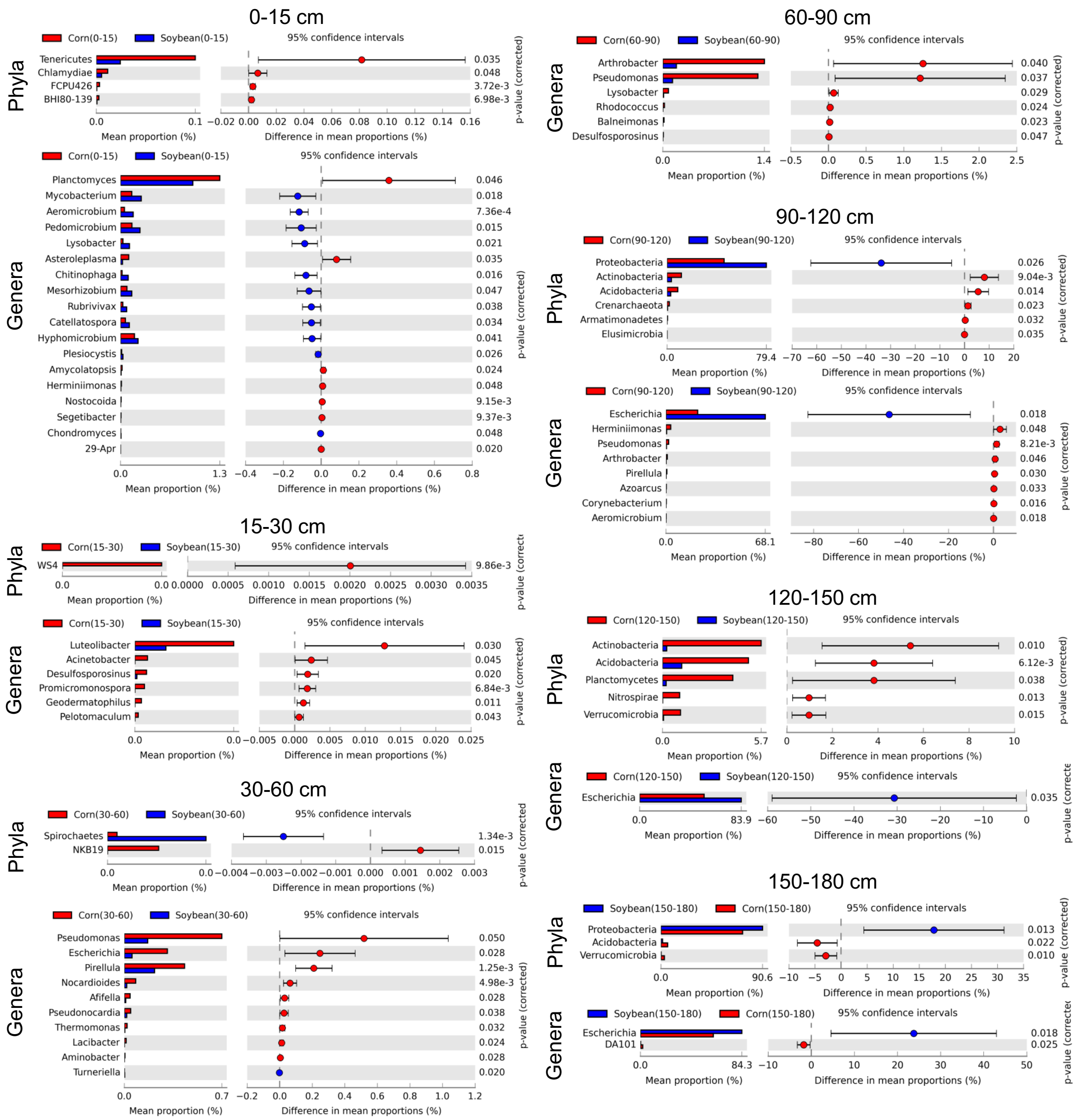
0-15 cm
Corn(0-15) Soybean(0-15) 95% confidence intervals
Phyla
Tenericutes 0.035
Chlamydiae 0.048
FCPU426 3.72e-3
BHI80-139 6.98e-3
Mean proportion (%)
Difference in mean proportions (%)
p-value (corrected)
Genera
Planctomyces 0.046
Mycobacterium 0.018
Aeromicrobium 7.36e-4
Pedomicrobium 0.015
Lysobacter 0.021
Asteroleplasma 0.035
Chitinophaga 0.016
Mesorhizobium 0.047
Rubrivivax 0.038
Catellatospora 0.034
Hyphomicrobium 0.041
Plesiocystis 0.026
Amycolatopsis 0.024
Herminiimonas 0.048
Nostocoida 9.15e-3
Segetibacter 9.37e-3
Chondromyces 0.048
29-Apr 0.020
15-30 cm
Corn(15-30) Soybean(15-30) 95% confidence intervals
Phyla
WS4 9.86e-3
Genera
Luteolibacter 0.030
Acinetobacter 0.045
Desulfosporosinus 0.020
Promicromonospora 6.84e-3
Geodermatophilus 0.011
Pelotomaculum 0.043
30-60 cm
Corn(30-60) Soybean(30-60) 95% confidence intervals
Phyla
Spirochaetes 1.34e-3
NKB19 0.015
Genera
Pseudomonas 0.050
Escherichia 0.028
Pirellula 1.25e-3
Nocardioides 4.98e-3
Afifella 0.028
Pseudonocardia 0.038
Thermomonas 0.032
Lacibacter 0.024
Aminobacter 0.028
Turneriella 0.020
60-90 cm
Corn(60-90) Soybean(60-90) 95% confidence intervals
Genera
Arthrobacter 0.040
Pseudomonas 0.037
Lysobacter 0.029
Rhodococcus 0.024
Balneimonas 0.023
Desulfosporosinus 0.047
90-120 cm
Corn(90-120) Soybean(90-120) 95% confidence intervals
Phyla
Proteobacteria 0.026
Actinobacteria 9.04e-3
Acidobacteria 0.014
Crenarchaeota 0.023
Armatimonadetes 0.032
Elusimicrobia 0.035
Genera
Escherichia 0.018
Herminiimonas 0.048
Pseudomonas 8.21e-3
Arthrobacter 0.046
Pirellula 0.030
Azoarcus 0.033
Corynebacterium 0.016
Aeromicrobium 0.018
120-150 cm
Corn(120-150) Soybean(120-150) 95% confidence intervals
Phyla
Actinobacteria 0.010
Acidobacteria 6.12e-3
Planctomycetes 0.038
Nitrospirae 0.013
Verrucomicrobia 0.015
Genera
Escherichia 0.035
150-180 cm
Soybean(150-180) Corn(150-180) 95% confidence intervals
Phyla
Proteobacteria 0.013
Acidobacteria 0.022
Verrucomicrobia 0.010
Genera
Escherichia 0.018
DA101 0.025

Fig. S6

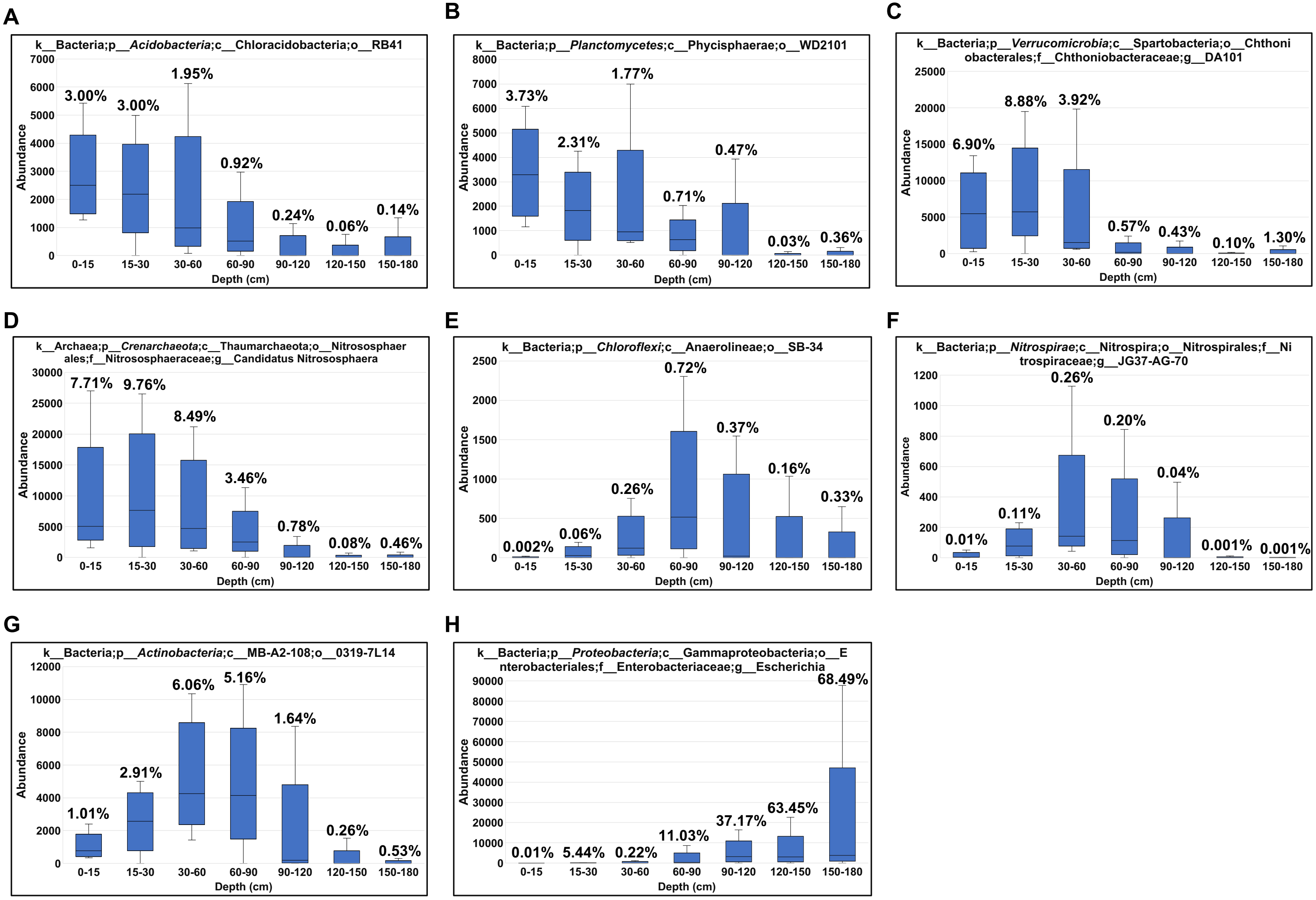
A
k__Bacteria;p__Acidobacteria;c__Chloracidobacteria;o__RB41
Abundance
Depth (cm)
3.00%
3.00%
1.95%
0.92%
0.24%
0.06%
0.14%
0-15
15-30
30-60
60-90
90-120
120-150
150-180
B
k__Bacteria;p__Planctomycetes;c__Phycisphaerae;o__WD2101
3.73%
2.31%
1.77%
0.71%
0.47%
0.03%
0.36%
C
k__Bacteria;p__Verrucomicrobia;c__Spartobacteria;o__Chthoniobacterales;f__Chthoniobacteraceae;g__DA101
6.90%
8.88%
3.92%
0.57%
0.43%
0.10%
1.30%
D
k__Archaea;p__Crenarchaeota;c__Thaumarchaeota;o__Nitrososphaerales;f__Nitrososphaeraceae;g__Candidatus Nitrososphaera
7.71%
9.76%
8.49%
3.46%
0.78%
0.08%
0.46%
E
k__Bacteria;p__Chloroflexi;c__Anaerolineae;o__SB-34
0.002%
0.06%
0.26%
0.72%
0.37%
0.16%
0.33%
F
k__Bacteria;p__Nitrospirae;c__Nitrospira;o__Nitrospirales;f__Nitrospiraceae;g__JG37-AG-70
0.01%
0.11%
0.26%
0.20%
0.04%
0.001%
0.001%
G
k__Bacteria;p__Actinobacteria;c__MB-A2-108;o__0319-7L14
1.01%
2.91%
6.06%
5.16%
1.64%
0.26%
0.53%
H
k__Bacteria;p__Proteobacteria;c__Gammaproteobacteria;o__Enterobacteriales;f__Enterobacteriaceae;g__Escherichia
0.01%
5.44%
0.22%
11.03%
37.17%
63.45%
68.49%

Fig. S7

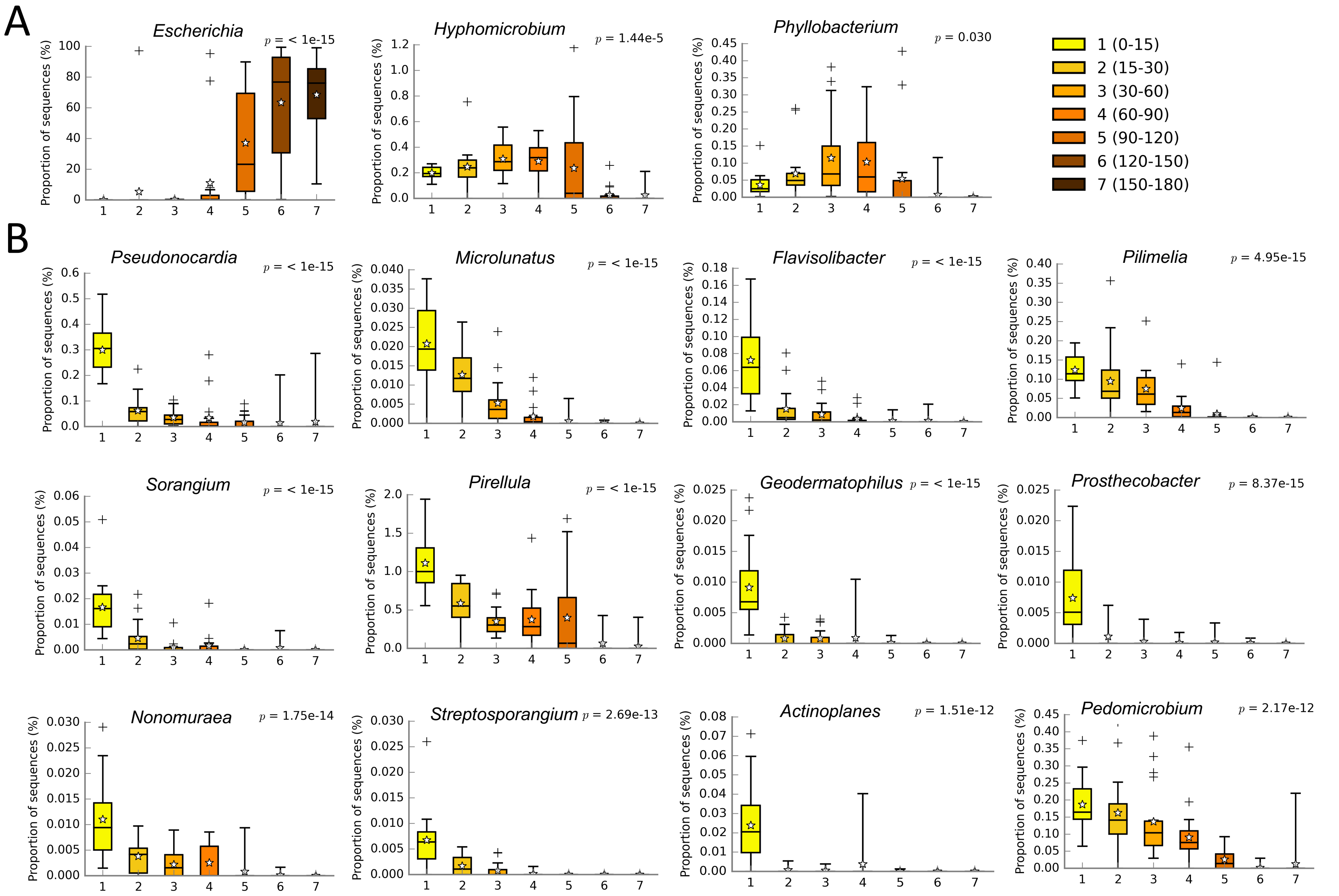
A
Escherichia
p = < 1e-15
Hyphomicrobium
p = 1.44e-5
Phyllobacterium
p = 0.030
1 (0-15)
2 (15-30)
3 (30-60)
4 (60-90)
5 (90-120)
6 (120-150)
7 (150-180)
B
Pseudonocardia
p = < 1e-15
Microlunatus
p = < 1e-15
Flavisolibacter
p = < 1e-15
Pilimelia
p = 4.95e-15
Sorangium
p = < 1e-15
Pirellula
p = < 1e-15
Geodermatophilus
p = < 1e-15
Prosthecobacter
p = 8.37e-15
Nonomuraea
p = 1.75e-14
Streptosporangium
p = 2.69e-13
Actinoplanes
p = 1.51e-12
Pedomicrobium
p = 2.17e-12
Proportion of sequences (%)

Fig. S8
